# Predicting hospital readmissions in older patients with heart failure with advanced bioinformatics tools: focus on the role of vulnerability and frailty

**DOI:** 10.1007/s11739-022-03099-2

**Published:** 2022-09-23

**Authors:** Marco Bertolotti, Carlotta Franchi, Giulia Lancellotti, Sara Mandelli, Chiara Mussi

**Affiliations:** 1 Division of Geriatrics, Department of Biomedical, Metabolic and Neural Sciences and Center for Gerontological Evaluation and Research, Ospedale Civile di Baggiovara, Università di Modena e Reggio Emilia, Via Giardini, 1355, 41026 Modena, MO Italy; 2 Department of Neuroscience, Istituto di Ricerche Farmacologiche Mario Negri IRCCS, Milan, Italy

Heart failure (HF) represents a leading cause for hospitalization in older patients [1]. Furthermore, its presence associates with a poorer clinical outcome, particularly when placed within a specific context of disease clustering [2].

Identification of risk factors and predisposing conditions for hospital readmission may be crucial, to prevent further hospitalization [3]. This should hopefully help in the personalization of disease management, allowing to focalize the implementation of preventive strategies on individuals at high risk for readmission.

A number of tools have been described in the literature on this regard. Different statistical approaches have been proposed, where the contribution of individual variables has been estimated with the aid of linear predictors or logistic regression.

Machine learning (ML) methods represent an innovative and interesting model. The possibility to incorporate a larger number of variables and to analyze nonlinear effects of variables allows for a better predictive power, when compared to conventional statistical techniques. Such approach has been validated, among others, in the prediction of adverse outcomes (mortality or hospitalization) in patients with heart failure with preserved ejection fraction [4].

In this issue of the Journal, a report by Polo Friz and coworkers explores the possible use of ML for predicting 30 day readmission after heart failure hospitalization [5]. The study, retrospective, has been run on a large cohort of patients older than age 65 (mean age 81), therefore highly representative of the population that we encounter every day in our Internal Medicine or Geriatrics wards.

The ML models were built taking into account a large set of clinical and biochemical data, most of which easily collectable from administrative databases.

Nearly 13% of the patients experienced a readmission in the 30 days following discharge, with no significant gender difference.

With the adoption of ML models for the prediction of subsequent hospitalization, ROC curves with an AUC of nearly 0.80 were obtained; such models apparently provided a better performance when compared with previously validated models, such as the LACE index. Analysis of the negative and positive predictive values likewise showed quite satisfactory results.

Interestingly, age was not statistically different in readmitted vs non-readmitted patients. This finding underlines the concept that chronological age in itself is a poor prognostic determinant. Furthermore, the present findings are consistent with the evidence obtained in younger population and this, once again, seems to suggest a relatively limited role of age per se in this context.

A deeper insight in the impact of individual variables of the dataset (SHAP analysis) showed that an increased risk of 30 day readmission was associated with of the number of previous hospitalizations and with the comorbidity burden; these parameters have been largely demonstrated to be predictors of adverse outcomes and they have often been included in the main scores of frailty [6].

Comorbidity is frequently, even if not invariably, associated with frailty; such combination indeed represents a relevant hallmark in the clinical picture of HF. It would be interesting to directly evaluate frailty in the population of Polo Friz’s work; to do this, other areas, which have not been included in this study, should be investigated, such as physical functioning and cognition, which represent a part of the complex definition of frailty.

Table 1 shows some of the clinical characteristics of the patients with or without HF in the cohort of REPOSI (REgistro POliterapie Società Italiana di Medicina Interna), a multicenter study collecting data from a large population of patients hospitalized in Internal Medicine or Geriatrics wards [2, 7].

**Table 1:** Age/gender distribution and clinical features of a population of older hospitalized patients of the REPOSI cohort from 2010 to 2019 [2, 7]

Variable	Population without HF *N* = 5755 (81.7%)	*N* Obs	Patients with HF *N* = 1290 (18.3%)	*N* Obs	P*
Gender		5753		1290	
Male	2832 (49.2)		616 (47.7)		< 0.0001
Female	2921 (50.8)		674 (52.2)		
Age—median (IQR)		5755		1290	
65–74	1784 (31.0)		237 (18.4)		< 0.0001
75–84	2515 (43.7)		554 (42.9)		
≥ 85	1456 (25.3)		499 (38.7)		
Barthel		4496		1009	
Total Dependence (%) (< 25)	385 (8.6)		105 (10.4)		< 0.0001
Severe Dependence (%) (25–49)	277 (6.2)		130 (12.9)		
Moderate Dependence (%) (50–74)	577 (12.8)		198 (19.6)		
Mild Dependence (%) (75–90)	819 (18.2)		254 (25.2)		
No Dependence (%) (91–100)	2438 (54.2)		322 (31.9)		
CIRS (Comorbidity)—median (IQR)	3 (1–4)	5755	4.0 (2.0–5.0)	1290	< 0.0001
CIRS (Severity)—median (IQR)	1.6 (1.4–1.8)		1.8 (1.6–2.0)	1290	< 0.0001
SBT		5027		1093	
Normal (0–4)	2131 (42.4)		339 (31.0)		< 0.0001
Possible Cognitive Impairment (5–9)	895 (17.8)		208 (19.0)		
Moderate Cognitive Impairment (10–19)	1425 (28.4)		396 (36.2)		
Severe Cognitive Impairment (20–28)	576 (11.5)		150 (13.7)		

*Chi-square for categorical variables; Wilcoxon test for continuous variables

As it can be seen, markers of dependence (here expressed by the Barthel index) and multimorbidity (CIRS-Comorbidity and CIRS-Severity) are significantly higher in HF patients. Furthermore, the cognitive profile (assessed using the SBT as a screening tool) is shifted toward more severe degrees of impairment in HF patients. Thus, HF patients appear to be older but, most importantly, more complex and with a more severe impairment of autonomy and cognition. The findings confirm previous evidence on the risk of readmission for all diseases [8].

Looking at these profiles, therefore, the use of sophisticated techniques taking into account different aspects of complexity, such as ML, may be expected to provide valuable information for the prediction of negative outcomes, in hospitalized older patients with HF. ML may be considered as complementary to other approaches aimed to characterize the different pattern of patient complexity and multimorbility, such as cluster analysis [2, 7].

Theoretically, the use of ML in heart disease patients might be extended to outpatients as well.

In a population of subjects attending a Cardiologic Geriatrics clinic, 358 patients out of 1704 (21.0%) had a clinical diagnosis of HF; among these, 29.2% were in NYHA stage 3 and 4.1% were in NYHA stage 4; that is, one third of them presented an advanced stage of the disease (Mussi C, Bertolotti M, unpublished data). This proportion appears to be rather high, when we consider that it refers to subjects with a relatively good degree of autonomy.

It appears absolutely reasonable that, particularly in the latter strata of HF patients, the adoption of ML strategies might help to identify the patients at higher risk of subsequent hospitalization for HF worsening. A predictive role for frailty assessment with regards to adverse outcomes in outpatients with HF has already been suggested [9].

The integration with telehealth/telemedicine devices might support such approach.

Proper prognostic stratification and subsequent identification of older patients at high risk of hospitalization/re-hospitalization, provided by ML or analogous methodologies, might prove helpful in the perspective of a “personalized” approach, allowing a more oriented allocation of resources. In this context, the consideration of complexity and the evaluation of vulnerability and/or frailty in its heterogeneous manifestations is crucial.
